# Association between metabolic dysfunction-associated steatotic liver disease and obstructive sleep apnea: a nationwide retrospective cohort study

**DOI:** 10.1038/s41598-026-46037-4

**Published:** 2026-03-30

**Authors:** Chan Ho Park, Sang Yi Moon, Bongjo Kim, Minkook Son

**Affiliations:** 1 Department of Internal Medicine, Dong-A University College of Medicine, 26 Daesingongwon-ro, Seo-gu, Busan, 49201 Republic of Korea; 2 Department of Physiology, Dong-A University College of Medicine, 32 Daeshingongwon-ro, Seo-gu, Busan, 49201 Republic of Korea; 3 Department of Data Sciences Convergence, Dong-A University Interdisciplinary Program, Busan, Republic of Korea; 4 DAU G-LAMP Project Group, Innovation Center for Atomic Science Dong-A University, Busan, Republic of Korea

**Keywords:** Diseases, Gastroenterology, Medical research, Risk factors

## Abstract

Metabolic dysfunction-associated steatotic liver disease (MASLD) and obstructive sleep apnea (OSA) share overlapping metabolic and inflammatory pathways, yet population-level evidence linking MASLD and incident OSA remains limited. Using a nationwide cohort of 265,452 Korean adults aged ≥ 40 years, we evaluated OSA risk across five mutually exclusive phenotypes defined by steatosis (fatty liver index [FLI] ≥ 30), cardiometabolic risk factors (CMRFs), and alcohol intake: no steatotic liver disease (SLD) without CMRFs, no SLD with CMRFs, MASLD without alcohol, MASLD with alcohol intake below the metabolic-associated alcohol-related liver disease (MetALD) threshold (men < 210 g/week, women < 140 g/week), and MetALD. During a mean follow-up of 9.5 years, 1,025 participants developed OSA. Compared with the reference group, adjusted hazard ratios (aHRs) for OSA were 1.18 (95% confidence intervals [CI] 0.93–1.50) in individuals with CMRFs alone, 1.46 (95% CI 1.12–1.91) in MASLD without alcohol, 1.52 (95% CI 1.17–1.98) in MASLD with alcohol, and 1.40 (95% CI 1.01–1.94) in MetALD. Model-based absolute risk differences (ARDs) at 9.5 years showed consistent patterns (+ 0.05%, + 0.14%, + 0.16%, and + 0.12%, respectively). Sensitivity analyses using stricter steatosis criteria (FLI ≥ 60 or hepatic steatosis index ≥ 36) demonstrated a clearer dose-related gradient, with progressively higher OSA risk across MASLD without alcohol, MASLD with alcohol, and MetALD. These findings highlight MASLD—particularly alcohol-associated phenotypes—as important risk markers for OSA and underscore the need for targeted screening and early intervention strategies in this increasingly prevalent population.

**Supplementary Information:**

The online version contains supplementary material available at 10.1038/s41598-026-46037-4.

## Introduction

OSA is a common and increasingly recognized sleep disorder characterized by recurrent episodes of partial or complete upper airway obstruction during sleep. These episodes result in intermittent hypoxia, sleep fragmentation, and sympathetic nervous system activation, contributing to a wide range of adverse health consequences. OSA is estimated to affect 9–38% of the global adult population, with higher prevalence among older adults, males, and individuals with obesity^1,2^. It is independently associated with increased risks of cardiovascular disease, stroke, insulin resistance, and all-cause mortality^3,4^. Despite its clinical significance, OSA remains underdiagnosed and undertreated, particularly in populations with coexisting metabolic disorders.

OSA has been closely linked to several metabolic conditions, with particularly strong associations observed with type 2 diabetes mellitus (T2DM), hypertension, and obesity. For instance, OSA has been shown to increase insulin resistance and worsen glycemic control in patients with T2DM through sympathetic overactivity and nocturnal hypoxemia^5^. Similarly, obesity—a major risk factor for both OSA and metabolic syndrome—exacerbates upper airway collapsibility and systemic inflammation^6^. Nonalcoholic fatty liver disease (NAFLD), the hepatic manifestation of metabolic syndrome, has also been increasingly associated with OSA. Previous studies have reported a bidirectional relationship between OSA and NAFLD, with intermittent hypoxia accelerating liver injury and hepatic steatosis, while fatty liver disease exacerbates cardiometabolic profiles that predispose to OSA^7–10^.

Recently, the term MASLD was introduced to replace NAFLD so as to better reflect the underlying pathophysiology and clinical heterogeneity of fatty liver disease. MASLD is diagnosed based on evidence of hepatic steatosis in the presence of at least one of five cardinal cardiometabolic risk factors (CMRFs): overweight/obesity, hypertension, hypertriglyceridemia, low HDL-C, or T2DM^11–13^. This redefinition has garnered substantial interest and has prompted renewed investigations into the role of MASLD in systemic metabolic and cardiovascular diseases^14^.

Despite this redefinition, few large-scale epidemiological studies have examined the association between MASLD and OSA. Given their shared metabolic origins and overlapping pathophysiological mechanisms, understanding this association has important clinical and public health implications. To address this gap, we conducted a nationwide retrospective cohort study using data from the Korean National Health Insurance Service (NHIS) to assess the risk of OSA in individuals with MASLD, with and without alcohol-related hepatic involvement. Furthermore, we sought to disentangle the independent and alcohol-modified components of MASLD by applying recently established MASLD–MetALD classifications. This approach enables a more refined assessment of how hepatic steatosis and alcohol exposure jointly shape OSA susceptibility.

## Results

### Baseline characteristics of the study population

**Fig. 1:**
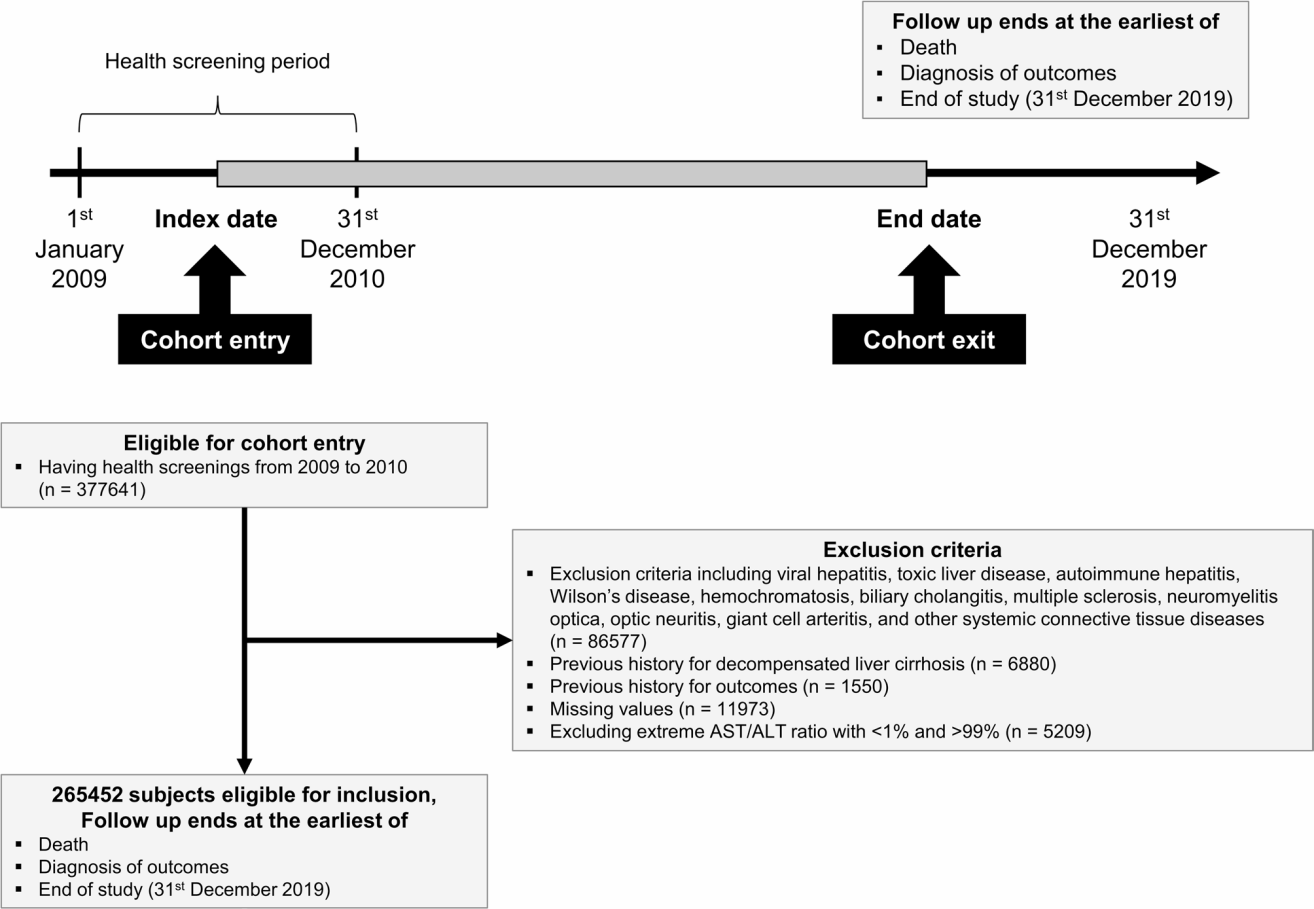
Flow diagram of the study population.

Baseline characteristics of the study population are presented in Table 1. After applying the exclusion criteria, a total of 265,452 participants were included in the final analysis (Fig. 1). Overall, 53.4% of participants were male. The mean age of participants was 58.9 ± 8.8 years. The mean body mass index (BMI) and waist circumference were 23.9 ± 3.0 kg/m^2^ and 81.5 ± 8.3 cm, respectively. Participants were classified into five mutually exclusive groups according to the presence of SLD and CMRFs: (1) no SLD without CMRF (*n* = 26,067), (2) no SLD with CMRFs (*n* = 138,580), (3) MASLD without alcohol consumption (*n* = 50,526), (4) MASLD with alcohol consumption (*n* = 38,207), and (5) MetALD (*n* = 12,072).

**Table 1:** Baseline characteristics of the study population.

Variables		No SLD without CMRF (*n* = 26067)	No SLD with CMRF (*n* = 138580)	MASLD without alcohol (*n* = 50526)	MASLD with alcohol (*n* = 38207)	MetALD (*n* = 12072)	*P*-value
Sex (%)	Male	12,701 (48.7)	58,832 (42.5)	23,788 (47.1)	34,979 (91.6)	11,555 (95.7)	< 0.001
	Female	13,366 (51.3)	79,748 (57.5)	26,738 (52.9)	3228 (8.4)	517 (4.3)	
Age (years)	Mean (SD)	55.9 (7.8)	59.7 (9.0)	61.5 (8.8)	56.9 (7.6)	56.2 (7.3)	< 0.001
Income level (%)	1st quartile	3564 (13.7)	20,425 (14.7)	7737 (15.3)	4107 (10.7)	1352 (11.2)	< 0.001
	2nd quartile	5586 (21.4)	29,294 (21.1)	10,624 (21.0)	6556 (17.2)	2195 (18.2)	
	3rd quartile	7264 (27.9)	40,019 (28.9)	15,644 (31.0)	10,805 (28.3)	3821 (31.7)	
	4th quartile	9653 (37.0)	48,842 (35.2)	16,521 (32.7)	16,739 (43.8)	4704 (39.0)	
Residence (%)	Rural	8280 (31.8)	48,551 (35.0)	19,575 (38.7)	11,848 (31.0)	4261 (35.3)	< 0.001
	Urban	17,787 (68.2)	90,029 (65.0)	30,951 (61.3)	26,359 (69.0)	7811 (64.7)	
Hypertension (%)		0 (0.0)	62,924 (45.4)	30,834 (61.0)	20,409 (53.4)	6771 (56.1)	< 0.001
Diabetes (%)		0 (0.0)	15,721 (11.3)	10,903 (21.6)	6864 (18.0)	2416 (20.0)	< 0.001
Dyslipidemia (%)		0 (0.0)	50,460 (36.4)	29,085 (57.6)	17,770 (46.5)	4864 (40.3)	< 0.001
Charlson comorbidity index (%)	0	16,397 (62.9)	65,574 (47.3)	18,895 (37.4)	19,962 (52.2)	6288 (52.1)	< 0.001
	1	6494 (24.9)	37,300 (26.9)	13,623 (27.0)	9874 (25.8)	3179 (26.3)	
	2	2253 (8.6)	18,728 (13.5)	8176 (16.2)	4536 (11.9)	1444 (12.0)	
	≥ 3	923 (3.5)	16,978 (12.3)	9832 (19.5)	3835 (10.0)	1161 (9.6)	
Body mass index (kg/m^2^**)**	Mean (SD)	20.9 (1.5)	23.0 (2.2)	26.5 (2.6)	25.7 (2.4)	25.4 (2.5)	< 0.001
Waist circumference (cm)	Mean (SD)	73.6 (5.9)	78.4 (6.3)	88.4 (6.4)	88.2 (6.0)	88.0 (6.3)	< 0.001
Systolic blood pressure (mmHg)	Mean (SD)	113.8 (10.7)	124.5 (15.2)	128.8 (15.1)	128.9 (14.4)	130.5 (14.6)	< 0.001
Diastolic blood pressure (mmHg)	Mean (SD)	71.0 (7.5)	76.8 (9.8)	79.2 (9.7)	80.6 (9.7)	81.7 (9.8)	< 0.001
Fasting blood glucose (mg/dL)	Mean (SD)	87.9 (7.4)	98.8 (21.9)	105.5 (28.5)	106.2 (27.6)	108.7 (29.8)	< 0.001
Total cholesterol (mg/dL)	Mean (SD)	189.6 (25.7)	198.4 (37.1)	207.7 (39.9)	204.1 (36.8)	202.8 (36.7)	< 0.001
Triglyceride (mg/dL)	Mean (SD)	81.8 (28.3)	107.1 (48.9)	186.0 (94.3)	188.5 (98.0)	200.3 (115.6)	< 0.001
HDL cholesterol (mg/dL)	Mean (SD)	62.2 (19.6)	56.0 (23.1)	50.4 (29.0)	50.8 (22.2)	53.6 (20.2)	< 0.001
LDL cholesterol (mg/dL)	Mean (SD)	111.3 (24.4)	121.7 (36.0)	121.9 (40.4)	116.9 (38.1)	110.5 (41.1)	< 0.001
Aspartate aminotransferase (U/L)	Mean (SD)	23.5 (9.7)	23.7 (8.2)	27.1 (14.6)	28.3 (14.9)	31.3 (23.9)	< 0.001
Alanine aminotransferase (U/L)	Mean (SD)	18.9 (11.0)	20.3 (10.1)	28.7 (18.2)	29.9 (17.9)	31.1 (21.8)	< 0.001
r-glutamyl transpeptidase (U/L)	Mean (SD)	22.1 (17.0)	22.1 (14.4)	39.1 (38.4)	60.3 (60.9)	86.5 (89.9)	< 0.001
Hemoglobin (g/dL)	Mean (SD)	13.5 (1.4)	13.5 (1.4)	13.9 (1.5)	14.8 (1.3)	14.9 (1.2)	< 0.001
Glomerular filtration rate (mL/min/1.73 m^**2**^**)**	Mean (SD)	81.7 (30.5)	78.7 (29.3)	75.7 (28.9)	78.3 (35.7)	80.6 (34.1)	< 0.001
Smoking (%)	Non-smoker	17,993 (69.0)	102,157 (73.7)	37,953 (75.1)	13,046 (34.1)	3068 (25.4)	< 0.001
	Ex-smoker	3585 (13.8)	19,734 (14.2)	7202 (14.3)	13,072 (34.2)	4078 (33.8)	
	Smoker	4489 (17.2)	16,689 (12.0)	5371 (10.6)	12,089 (31.6)	4926 (40.8)	
Alcohol consumption (%)		9676 (37.1)	42,849 (30.9)	226 (0.4)	38,207 (100.0)	12,072 (100.0)	< 0.001
Amount of alcohol consumption (g/week)	Mean (SD)	41.3 (97.4)	35.4 (94.7)	0.0 (0.0)	87.0 (55.1)	287.5 (70.4)	< 0.001
Regular exercise (%)	No	17,435 (66.9)	94,884 (68.5)	37,608 (74.4)	20,705 (54.2)	7175 (59.4)	< 0.001
	1–2 times/week	5297 (20.3)	24,704 (17.8)	7381 (14.6)	11,333 (29.7)	2953 (24.5)	
	3–4 times/week	2087 (8.0)	11,475 (8.3)	3307 (6.5)	4116 (10.8)	1265 (10.5)	
	5 times/week	1248 (4.8)	7517 (5.4)	2230 (4.4)	2053 (5.4)	679 (5.6)	
Fatty liver index	Mean (SD)	7.9 (5.5)	15.1 (7.7)	49.1 (15.1)	52.9 (16.4)	57.7 (17.6)	< 0.001

Notable differences in demographic and metabolic characteristics were observed across the groups. The MASLD with alcohol and MetALD groups exhibited the highest proportion of males (91.6% and 95.7%, respectively) and the youngest mean ages (56.9 ± 7.6 years and 56.2 ± 7.3 years). In contrast, the MASLD without alcohol group had the highest mean BMI (26.5 ± 2.6 kg/m^2^) and waist circumference (88.4 ± 6.4 cm). The prevalence of hypertension, diabetes, and dyslipidemia was substantially higher in all MASLD phenotypes than in the no-SLD groups. Additionally, liver enzymes (aspartate aminotransferase [AST], alanine aminotransferase [ALT], and γ-glutamyl transferase [γ-GTP]), triglycerides, and the Charlson Comorbidity Index (CCI) were elevated among MASLD groups, whereas HDL-C levels were lower, reflecting a more adverse metabolic profile consistent with metabolic dysfunction-associated liver disease.

### Association between SLD and OSA

As shown in Table 2, during a mean follow-up of 9.5 years, 1025 participants developed OSA. Compared with the reference group (no SLD and no CMRF), the adjusted HR for OSA in individuals with CMRFs alone was not significant (aHR 1.18; 95% CI 0.93–1.50; *p* = 0.179). In contrast, all MASLD groups demonstrated significantly higher OSA risk. The aHRs were 1.46 (95% CI 1.12–1.91; *p* = 0.006) for MASLD without alcohol, 1.52 (95% CI 1.17–1.98; *p* = 0.002) for MASLD with alcohol below MetALD thresholds, and 1.40 (95% CI 1.01–1.94; *p* = 0.042) for MetALD. Model-based ARDs showed similar patterns (MASLD without alcohol: +0.14%; MASLD with alcohol: +0.16%; MetALD: +0.12%).

**Table 2:** Association between SLD and OSA.

Group	Number	Events	Follow-up duration (person-years)	Incidence rate (per 1000 person-years)	Crude HR (95% CIs, *P*-value)	Adjusted HR (95% CIs, *P*-value)*	Absolute risk difference [95% CIs]**
No SLD without CMRF	26,067	79	248,970.6	0.32	1 (Reference)	1 (Reference)	Reference
No SLD with CMRF	138,580	428	13,15,323.0	0.33	1.03 (0.81–1.30, *p* = 0.839)	1.18 (0.93–1.50, *p* = 0.179)	0.05% [-0.02% to 0.13%]
MASLD without alcohol	50,526	194	479,543.9	0.40	1.27 (0.98–1.65, *p* = 0.070)	1.46 (1.12–1.91, *p* = 0.006)	0.14% [0.04% to 0.23%]
MASLD with alcohol	38,207	251	360,506.2	0.70	2.20 (1.71–2.84, *p* < 0.001)	1.52 (1.17–1.98, *p* = 0.002)	0.16% [0.06% to 0.24%]
MetALD	12,072	73	113,750.7	0.64	2.03 (1.48–2.79, *p* < 0.001)	1.40 (1.01–1.94, *p* = 0.042)	0.12% [0% to 0.23%]
MASLD without alcohol	50,526	194	479,543.9	0.40	1 (Reference)	1 (Reference)	Reference
MASLD with alcohol	38,207	251	360,506.2	0.70	1.72 (1.43–2.08, *p* < 0.001)	1.00 (0.82–1.24, *p* = 0.964)	0.02% [-0.06% to 0.11%]
MetALD	12,072	73	113,750.7	0.64	1.59 (1.21–2.08, *p* = 0.001)	0.92 (0.69–1.22, *p* = 0.556)	-0.02% [-0.13% to 0.12%]

*The model was adjusted for age, sex, income level, residence area, Charlson comorbidity index, hemoglobin level, glomerular filtration rate, and smoking and regular exercise status.

**Absolute risk differences were calculated based on cumulative incidence estimates from the fully adjusted Cox models.

### Dose–response relationship between the number of CMRFs and OSA risk

Among individuals without SLD, a clear dose–response pattern in OSA risk according to the number of CMRFs was not observed. Compared with the reference group (no SLD without CMRF), adjusted HRs varied modestly across CMRF strata, but none reached statistical significance (one CMRF: aHR 1.24, 95% CI 0.95–1.63; two CMRFs: aHR 1.06, 95% CI 0.81–1.40; ≥3 CMRFs: aHR 1.26, 95% CI 0.95–1.68). In contrast, individuals with MASLD (aHR 1.49, 95% CI 1.17–1.91; *p* = 0.001) and MetALD (aHR 1.40, 95% CI 1.01–1.94; *p* = 0.044) showed significantly higher risks of OSA compared with the reference group (Table 3).

**Table 3:** Association between CMRF burden, steatotic phenotypes, and OSA.

Group	Number	Events	Follow-up duration (person-years)	Incidence rate (per 1000 person-years)	Crude HR (95% CIs, *P*-value)	Adjusted HR (95% CIs, *P*-value)*
No SLD without CMRF	26,067	79	248,970.6	0.32	1 (Reference)	1 (Reference)
No SLD with 1 CMRF	43,976	156	418,482.0	0.37	1.17 (0.90–1.54, *p* = 0.244)	1.24 (0.95–1.63, *p* = 0.114)
No SLD with 2 CMRF	50,999	142	484,116.0	0.29	0.92 (0.70–1.22, *p* = 0.574)	1.06 (0.81–1.40, *p* = 0.662)
No SLD with 3–4 CMRF	43,605	130	412,724.9	0.31	0.99 (0.75–1.31, *p* = 0.958)	1.26 (0.95–1.68, *p* = 0.111)
MASLD	88,733	445	840,050.1	0.53	1.67 (1.32–2.12, *p* < 0.001)	1.49 (1.17–1.91, *p* = 0.001)
MetALD	12,072	73	113,750.7	0.64	2.03 (1.48–2.79, *p* < 0.001)	1.40 (1.01–1.94, *p* = 0.044)

*The model was adjusted for age, sex, income level, residence area, Charlson comorbidity index, hemoglobin level, glomerular filtration rate, and smoking and regular exercise status.

### Sensitivity analyses

**Fig. 2:**
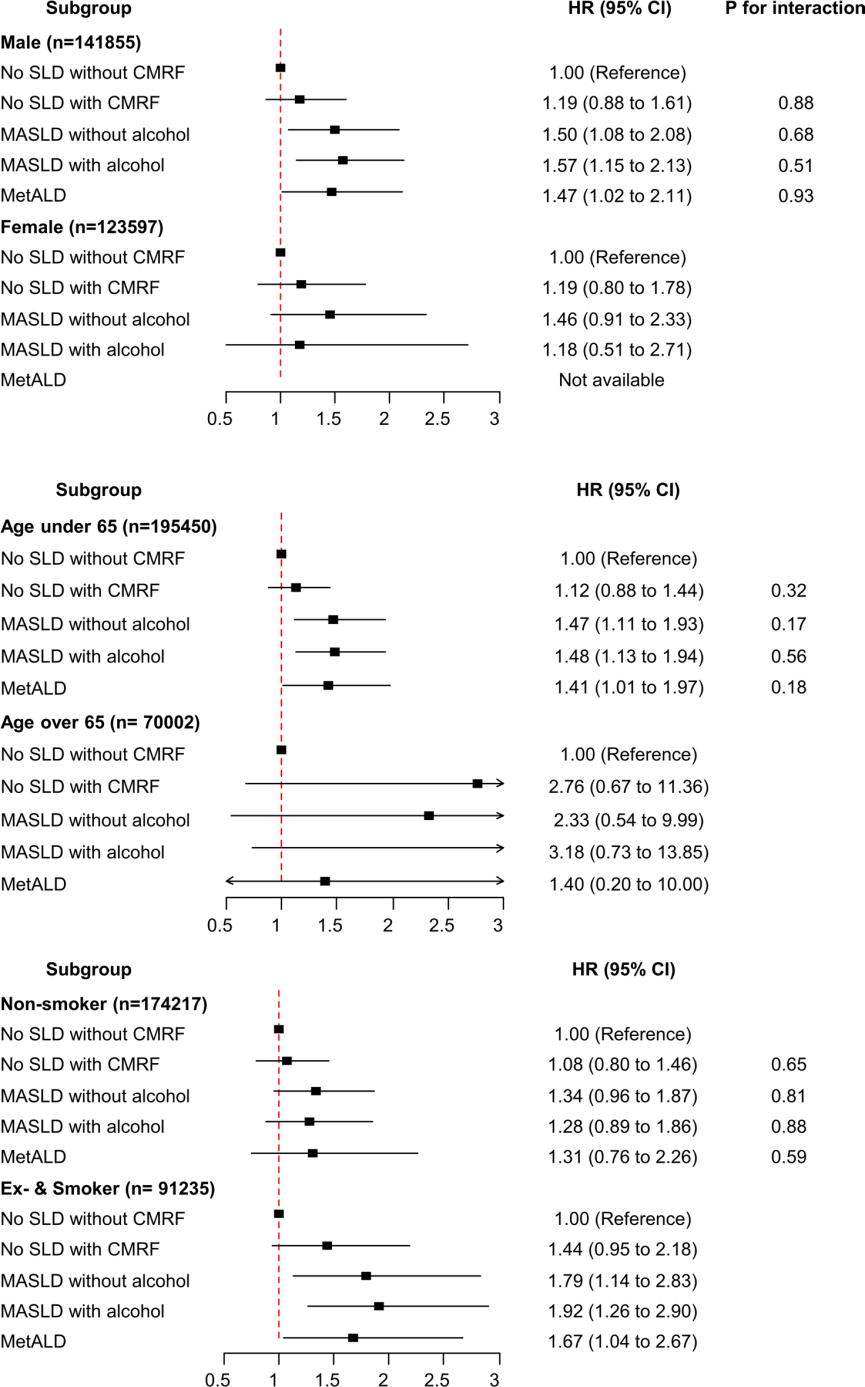
Stratified and interaction analyses of the association between metabolic dysfunction-associated steatotic liver disease (MASLD) and incident obstructive sleep apnea (OSA). *Adjusted hazard ratios (aHRs) and 95% confidence intervals are shown across predefined subgroups, including sex, age, and smoking status. Estimates were derived from fully adjusted Cox proportional hazards models. P values for interaction represent effect modification between MASLD and each subgroup variable.

Sensitivity analyses using stricter steatosis definitions yielded consistent results (Supplementary Table 2). Using FLI ≥ 60, a similar pattern of elevated risk across MASLD phenotypes was observed (aHR 1.70, 1.61, and 1.62, respectively). Similarly, analyses based on hepatic steatosis index (HSI) ≥ 36 showed increasing OSA risk across metabolic-alcohol phenotypes (aHR 1.67, 1.64, and 2.09, respectively). These results reinforce the robustness of the primary findings. In stratified analyses, the association between MASLD and incident OSA was generally consistent across sex, age, and smoking status, with no statistically significant effect modification observed (Fig. 2).

### Graphical representation of risk gradients

**Fig. 3:**
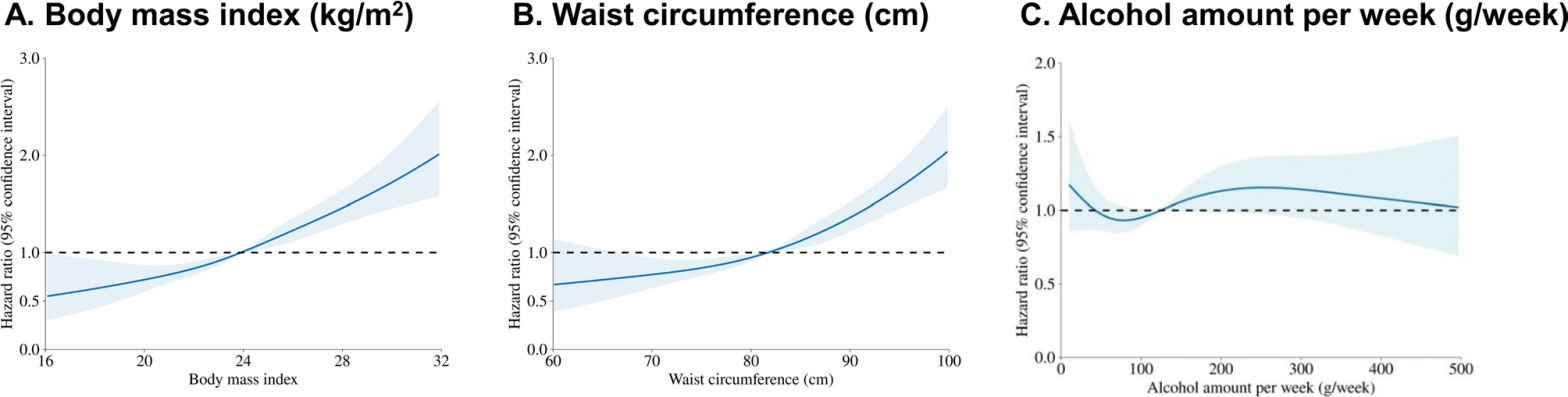
Restricted cubic spline of hazard ratio with 95% confidence intervals for OSA. *The model was adjusted for age, sex, income level, residence area, Charlson comorbidity index, hemoglobin level, glomerular filtration rate, and smoking and regular exercise status.

Figure 3 presents restricted cubic spline analyses demonstrating positive associations between BMI, waist circumference, and OSA risk. The alcohol–OSA association exhibited a J-shaped pattern, with an apparent decline at very high intake due to sparse data and wider confidence intervals.

**Fig. 4:**
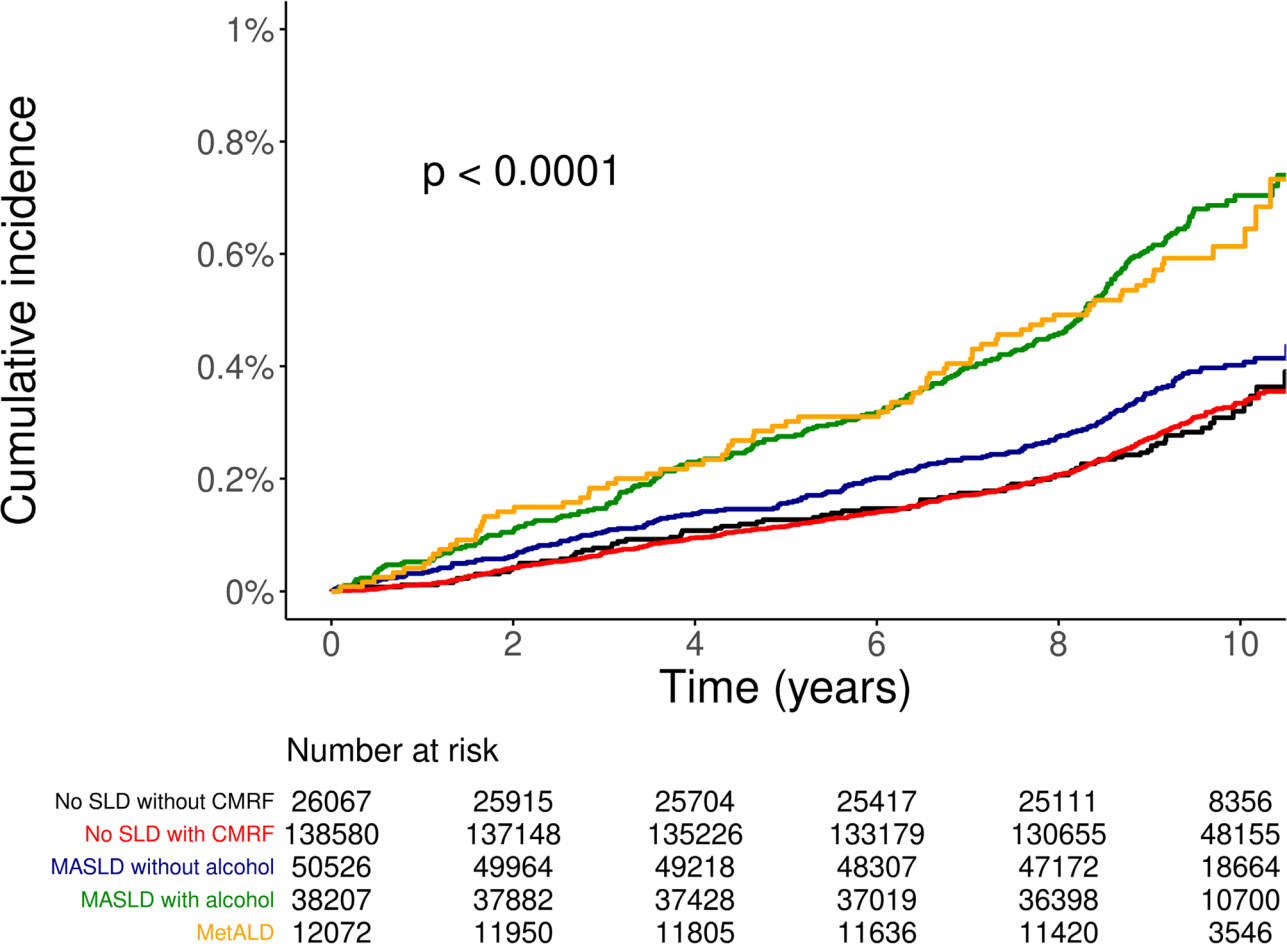
Kaplan–Meier curves for the association between SLD and OSA.

The Kaplan–Meier analysis (Fig. 4) illustrated clear visual differences in the cumulative incidence of OSA across the five exposure groups. The MASLD with alcohol and MetALD groups displayed the highest cumulative incidence throughout follow-up, followed by the MASLD without alcohol group. Both MASLD phenotypes showed higher incidence trajectories than the two non-SLD groups, with the lowest incidence observed in the reference category. Log-rank testing indicated statistically significant differences in OSA incidence across the groups (*p* < 0.001).

## Discussion

In this large, nationwide cohort of Korean adults, we found that MASLD was associated with a significantly higher risk of incident OSA, whereas CMRFs in the absence of steatosis did not confer a statistically meaningful increase in risk. Although modest increases in OSA incidence were observed with a greater number of CMRFs among individuals without SLD, the lack of a clear dose–response pattern suggests that CMRFs alone may not fully capture the metabolic phenotype linked to elevated OSA susceptibility, consistent with prior research highlighting the multifactorial nature of OSA beyond traditional metabolic predictors^1–6^. In contrast, all MASLD and MetALD phenotypes exhibited significantly higher OSA risk, supporting the notion that the metabolic–hepatic phenotype identified by MASLD may serve as a more clinically informative marker of OSA risk than CMRFs alone^7–10,15−17^.

Alcohol intake appeared to further modify OSA risk among individuals with MASLD. Although differences between alcohol consumption strata did not reach statistical significance in subgroup analyses, point estimates suggested a tendency toward higher OSA risk with increasing levels of alcohol exposure. This observation is consistent with established evidence indicating that alcohol increases upper-airway collapsibility and exacerbates sleep-disordered breathing^18–20^. Several factors may explain the lack of statistical significance, including reduced statistical power in the highest alcohol category (MetALD) and the well-known tendency for under-reporting of alcohol consumption in large-scale cohorts. Importantly, sensitivity analyses using stricter definitions of steatosis demonstrated a clearer alcohol-related gradient, reinforcing the possibility that alcohol may act as an additive contributor to OSA risk in the presence of metabolic liver disease. Notably, the HSI-based definition produced a higher effect estimate for MetALD (aHR 2.09), suggesting that indices with stronger metabolic weighting may better capture OSA-related risk among heavy drinkers.

The restricted cubic spline analyses further illustrated nonlinear associations between continuous metabolic indices and OSA risk. BMI and waist circumference showed strong, monotonic increases in risk, in line with extensive epidemiologic evidence identifying obesity as the most prominent modifiable determinant of OSA^21–22^. FLI demonstrated a positive association as well, but its interpretation warrants caution because FLI incorporates several metabolic components—including BMI, waist circumference, and triglycerides—that are themselves strongly related to OSA risk. Thus, the spline findings likely reflect composite metabolic burden rather than the isolated effect of hepatic steatosis. Nonetheless, the consistent elevation of OSA risk across MASLD phenotypes highlights the potential value of MASLD as a risk-identifying phenotype, echoing prior work on MASLD-related systemic inflammation, oxidative stress, and metabolic dysfunction^6,9,15,23–27^.

Kaplan–Meier analyses tended to show higher cumulative OSA incidence across the metabolic–hepatic phenotypes, with MASLD with alcohol and MetALD showing the steepest incidence curves over time. Model-based ARDs further supported these findings by providing a clinically interpretable measure of excess risk attributable to each phenotype. Although ARDs were modest, their consistency across multiple analytic approaches suggests that these estimates reflect true population-level associations. This pattern aligns with the high global burden of OSA^1,2^ and the increasing prevalence of MASLD across diverse populations^11–14^.

Several mechanisms may underlie the association between MASLD and OSA. Intermittent hypoxia, a hallmark of OSA, promotes hepatic steatosis progression, inflammation, oxidative stress, and fibrosis^7–9,15,25,26,28^, while metabolic dysfunction characteristic of MASLD may exacerbate ventilatory instability and impair neuromuscular control of the upper airway^6,27,29^. These overlapping pathophysiological pathways suggest a bidirectional relationship between OSA and metabolic liver disease, as proposed in recent reviews and expert consensus reports^15,29^. This notion is also supported by emerging work on liver–brain circadian signaling pathways^30^.

This study has notable strengths, including a large and nationally representative sample, standardized health screening data, rigorous phenotypic classification based on recent MASLD–MetALD consensus definitions^11–13^, and multiple complementary analytic approaches incorporating HRs, ARDs, and spline analyses. The long follow-up period and robust sensitivity analyses further enhance confidence in the observed associations. Notably, the association between MASLD and OSA remained consistent across multiple clinically relevant subgroups, supporting the robustness of our findings and mitigating concerns regarding residual confounding or exposure misclassification. However, limitations include reliance on surrogate indices rather than imaging for steatosis assessment, claims-based OSA diagnoses that likely underestimate mild cases, potential under-reporting of alcohol consumption, and residual confounding from unmeasured factors.

In conclusion, MASLD was associated with a higher risk of developing OSA, and alcohol exposure may further amplify this risk, particularly when steatosis is more severe or metabolic dysfunction is pronounced. These findings underscore the importance of recognizing MASLD—especially alcohol-associated phenotypes—as clinically relevant risk markers for OSA and support targeted screening strategies in this high-risk population.

## Methods

### Study population and design

This retrospective cohort study was based on data from the NHIS, a nationwide claims database that provides universal healthcare coverage. The database includes eligibility information, diagnoses (based on ICD-10 code), prescriptions, mortality data, and biennial national health screening results for adults aged ≥ 40 years. Health screening data comprise anthropometric measurements, laboratory test results, and standardized self-reported questionnaires on lifestyle behaviors (e.g., alcohol consumption, smoking and regular exercise status), with all components subject to national quality control standards. Using this dataset, we initially identified 377,641 individuals who underwent health examinations between January 1, 2009, and December 31, 2010. Among them, 86,577 were excluded due to diagnoses of viral hepatitis, autoimmune hepatitis, toxic liver disease, Wilson’s disease, biliary cholangitis, or other autoimmune and systemic inflammatory conditions with potential to cause chronic liver disease or confound the hepatic phenotype (detailed criteria provided in Supplementary Table 1). An additional 1,550 individuals were excluded due to a prior diagnosis of OSA. Further exclusions were applied for decompensated liver cirrhosis (*n* = 6,880), incomplete health screening data (*n* = 11,973), and extreme values of AST/ALT ratio (*n* = 5,209). After applying these criteria, a total of 265,452 individuals remained and were followed from the date of their baseline examination until the earliest of the following: diagnosis of OSA, death, or the end of the study period (December 31, 2019) (Fig. 1).

### Group classification using CMRFs and FLI

The CMRFs used in this study were defined based on the five components of the diagnostic criteria for MASLD. These included (1) a BMI ≥ 23 kg/m^2^ or a waist circumference ≥ 90 cm for men and ≥ 85 cm for women, as specified in the Korean Obesity Study Guidelines; (2) a fasting blood glucose level ≥ 100 mg/dL or the diagnosis and treatment of T2DM; (3) a blood pressure reading of ≥ 130/85 mmHg or the use of antihypertensive medication; (4) a serum triglyceride level ≥ 150 mg/dL or the use of lipid-lowering therapy; and (5) an HDL-C level ≤ 40 mg/dL for men or ≤ 50 mg/dL for women, or the use of lipid-lowering therapy. The presence of at least one of the above criteria qualified an individual as having CMRF.

SLD was defined using FLI, a validated non-invasive marker calculated from BMI, waist circumference, triglyceride levels, and γ-GTP. A threshold of FLI ≥ 30 was used to define hepatic steatosis, in accordance with established epidemiological practice. Based on the presence or absence of SLD, CMRFs, and alcohol consumption, participants were categorized into five mutually exclusive groups: (1) individuals with no SLD and no CMRFs (FLI < 30 and no CMRFs); (2) individuals with no SLD but with one or more CMRFs (FLI < 30 and ≥ 1 CMRF); (3) individuals with MASLD without alcohol consumption (FLI ≥ 30 and ≥ 1 CMRF); and (4) individuals classified as MASLD with alcohol consumption below the MetALD threshold (FLI ≥ 30 and ≥ 1 CMRF, with alcohol intake < 210 g/week for men and < 140 g/week for women); and (5) individuals classified as MetALD, defined as hepatic steatosis with alcohol intake at or above 210 g/week for men and 140 g/week for women. This classification follows the recent international consensus definitions proposed by Eslam et al.^11^ and Rinella et al.^13^.

To further investigate the relationship between the number of accompanying CMRFs and the risk of OSA, the following subclassification was applied to non-SLD individuals according to CMRF burden, while MASLD and MetALD were analyzed as separate steatotic phenotypes: (1) those without SLD and without any CMRFs; (2) those without SLD but with one CMRF; (3) those without SLD and two CMRFs; (4) those without SLD and three to four CMRFs; and (5) those with MASLD, regardless of the number of CMRFs.

### Outcome definition

OSA was defined using ICD-10 code G47.3, requiring (1) at least one inpatient or outpatient claim with G47.3 and (2) at least one additional G47.3-coded outpatient visit within 12 months to improve diagnostic specificity, consistent with the operational definition used in the Supplementary Table 1.

#### Covariates

Baseline demographic and clinical variables included age, sex, household income (quartile), region (urban vs. rural), and CCI. Comorbidities such as hypertension, T2DM, and dyslipidemia were identified using ICD-10 codes and prescription records. Laboratory and clinical measures included BMI, waist circumference, systolic and diastolic blood pressure, fasting glucose, lipid profile, liver enzymes (AST, ALT, γ-GTP), hemoglobin, and estimated glomerular filtration rate (eGFR). Lifestyle behaviors—including alcohol consumption, smoking and regular exercise status—were assessed through standardized self-reported questionnaires. Covariates were selected based on prior epidemiologic evidence linking demographic, lifestyle, and metabolic parameters with OSA risk, including age, sex, socioeconomic status, smoking and regular exercise status^21,22^. Alcohol consumption was additionally included to minimize confounding related to alcohol-associated liver injury, given its established effects on upper-airway collapsibility and its biological interaction with hepatic steatosis. Hemoglobin, eGFR, and the CCI were incorporated to account for systemic disease burden. To evaluate the robustness of our findings, supplementary models were constructed with further adjustment for BMI and individual cardiometabolic comorbidities, which allowed assessment of potential residual confounding arising from metabolic risk factors that are also components of MASLD diagnostic criteria. Detailed results of these supplementary models are presented in Supplementary Table 2.

### Statistical analysis

Baseline characteristics were summarized as means (standard deviations) or frequencies (percentages), and between-group differences were evaluated using ANOVA or the chi-square test, as appropriate. Cox proportional hazards regression models were used to estimate HRs and 95% CIs for incident OSA, with the “no SLD without CMRF” group serving as the reference category. Multivariable models were adjusted for age, sex, income level, region, CCI, hemoglobin level, eGFR, smoking and regular exercise status.

To enhance clinical interpretability, model-based absolute risk and ARDs were additionally calculated. Absolute risk at the mean follow-up time of 9.5 years was estimated using the riskRegression and pec packages in R, holding covariates at their sample means to allow comparability across exposure groups. ARDs were defined as the difference in predicted absolute risk relative to the reference group. 95% CIs for ARDs were obtained through 500 nonparametric bootstrap resamples, in which the Cox model was refitted and absolute risks were recomputed for each resampled dataset.

Kaplan–Meier curves were generated to compare cumulative OSA incidence between groups, and differences were evaluated using the log-rank test. Restricted cubic spline regression was used to explore potential nonlinear associations between continuous metabolic parameters—including FLI—and OSA risk. Sensitivity analyses were performed to examine the robustness of the findings: first, using a stricter FLI cutoff (≥ 60), and second, redefining steatosis using a HSI ≥ 36. In additional sensitivity analyses, we performed stratified analyses according to age group, sex, obesity and central obesity, smoking status, and alcohol consumption. Interaction terms between MASLD and each subgroup variable were included in the fully adjusted Cox models to assess potential effect modification and the consistency of the association. All analyses were performed using SAS version 9.4 and R version 4.3.0. Statistical significance was defined as a two-sided *p* < 0.05.

## Supplementary Information

Below is the link to the electronic supplementary material.

Supplementary Material 1

## Data Availability

The datasets generated and/or analyzed during the current study are available from the corresponding author, C.H.P., upon reasonable request.
