# Supplementary material for: Association between metabolic dysfunction-associated steatotic liver disease and obstructive sleep apnea: a nationwide retrospective cohort study
Source: Sci Rep. 2026 Mar 30;16:10572. doi: 10.1038/s41598-026-46037-4 (PMC13039169; doi:10.1038/s41598-026-46037-4)
Supplement: Supplementary file 1 — Supplementary Material 1 [file 41598_2026_46037_MOESM1_ESM.docx]

**Supplementary Table 1. The operational definition of variables**

|  | **ICD-10 codes** | **Definition** |
| --- | --- | --- |
| **Exclusion criteria** | | |
| Viral hepatitis | B15, B16, B17, B18, B19 | Admission ≥ 1 or outpatient clinic ≥ 1 |
| Toxic liver disease | K71 | Admission ≥ 1 or outpatient clinic ≥ 1 |
| Autoimmune hepatitis | K754 | Admission ≥ 1 or outpatient clinic ≥ 1 |
| Wilson’s disease | E830 | Admission ≥ 1 or outpatient clinic ≥ 1 |
| Hemochromatosis | E831 | Admission ≥ 1 or outpatient clinic ≥ 1 |
| Biliary cholangitis | K743, K744, K745 | Admission ≥ 1 or outpatient clinic ≥ 1 |
| Multiple sclerosis | G35 | Admission ≥ 1 or outpatient clinic ≥ 1 |
| Neuromyelitis optica | G36 | Admission ≥ 1 or outpatient clinic ≥ 1 |
| Optic neuritis | H46 | Admission ≥ 1 or outpatient clinic ≥ 1 |
| Giant cell arteritis | M315, M316 | Admission ≥ 1 or outpatient clinic ≥ 1 |
| Other systemic connective tissue diseases | M353 | Admission ≥ 1 or outpatient clinic ≥ 1 |
| Decompensated liver cirrhosis | R17, R18, K72, I850, I864, I983, K767 | Admission ≥ 1 or outpatient clinic ≥ 1 |
| **Outcomes** | | |
| Obstructive sleep apnea | G473 | Admission ≥ 1 or outpatient clinic ≥ 1 with at least one more OSA-related follow-up visit within 1year after the initial diagnosis |
| **Hepatic steatosis assessment** | | |
| Fatty liver index (FLI) | FLI = 1/(1+e^-x^)×100 where x = 0.953×ln(triglyceride, mg/dL) + 0.139×BMI (kg/m^2^) + 0.718×ln(γ-GTP, U/L) + 0.053×waist circumference (cm) – 15.745  **Cut-off values** < 30, low-risk; 30 to <60, intermediate-risk; ≥ 60, high-risk  **SLD definition**  Main analysis ≥ 30, Sensitivity analysis ≥ 60 | |
| Hepatic steatosis index (HSI) | HSI = 8×ALT/AST ratio + BMI (kg/m^2^) (+2, if diabetes; +2, if female)  **Cut-off values** < 30, ruled out; >36, highly likely  **SLD definition**  Sensitivity analysis > 36 | |
| **Cardiometabolic risk factors** | | |
| Hypertension | I10, I11 | Admission ≥ 1 or outpatient clinic ≥ 1 with any anti-hypertensive medication |
| Diabetes | E11, E12, E13, E14 | Admission ≥ 1 or outpatient clinic ≥ 1 with any anti-diabetic medication |
| Dyslipidemia | E78 | Admission ≥ 1 or outpatient clinic ≥ 1 with any lipid-lowering medication |

**Supplementary Table 2. Sensitivity analysis between SLD and OSA with different SLD definitions**

| **Group** | **Number** | **Events** | **Follow-up duration (person-years)** | **Incidence rate  (per 1000 person-years)** | **Crude HR  (95% CIs, P-value)** | **Adjusted HR  (95% CIs, P-value)** |
| --- | --- | --- | --- | --- | --- | --- |
| **Fatty liver index over 60** |  |  |  |  |  |  |
| No SLD without CMRF | 26067 | 79 | 248970.61 | 0.32 | 1  (Reference) | 1  (Reference) |
| No SLD with CMRF | 210474 | 762 | 1996754.47 | 0.38 | 1.20  (0.95-1.52, p=0.118) | 1.26  (1.00-1.59, p=0.052) |
| MASLD without alcohol | 11575 | 57 | 109130.08 | 0.52 | 1.65  (1.17-2.32, p=0.004) | 1.70  (1.20-2.41, p=0.003) |
| MASLD with alcohol | 12180 | 89 | 114615.15 | 0.78 | 2.46  (1.82-3.33, p<0.001) | 1.61  (1.18-2.20, p=0.003) |
| MetALD | 5156 | 38 | 48624.05 | 0.78 | 2.47  (1.68-3.64, p<0.001) | 1.62  (1.09-2.40, p=0.017) |
| **HSI** |  |  |  |  |  |  |
| No SLD without CMRF | 25936 | 79 | 247691.04 | 0.32 | 1  (Reference) | 1  (Reference) |
| No SLD with CMRF | 186049 | 660 | 1757953.23 | 0.38 | 1.18  (0.93-1.49, p=0.167) | 1.20  (0.95-1.52, p=0.131) |
| MASLD without alcohol | 35110 | 143 | 337614.71 | 0.42 | 1.32  (1.01-1.74, p=0.046) | 1.67  (1.26-2.21, p<0.001) |
| MASLD with alcohol | 14701 | 108 | 139960.08 | 0.77 | 2.42  (1.81-3.24, p<0.001) | 1.64  (1.22-2.21, p=0.001) |
| MetALD | 3501 | 35 | 33356.33 | 1.05 | 3.30  (2.21-4.91, p<0.001) | 2.09  (1.40-3.14, p<0.001) |
